# Interpersonal Distance in the SARS-CoV-2 Crisis

**DOI:** 10.1177/0018720820956858

**Published:** 2020-09-09

**Authors:** Robin Welsch, Heiko Hecht, Lewis Chuang, Christoph von Castell

**Affiliations:** 1 9183 Ludwig-Maximilians-Universität München, Germany; 2 9182 Johannes Gutenberg-Universität Mainz, Germany

**Keywords:** SARS-CoV-2, interpersonal distance, proxemics, discomfort

## Abstract

**Background:**

Mandatory rules for social distancing to curb the SARS-CoV-2 pandemic require individuals to maintain a critical interpersonal distance above 1.5 m. However, this contradicts our natural preference, which is closer to 1 m for non-intimate encounters, for example, when asking a stranger for directions.

**Objective:**

This review addresses how humans typically regulate interpersonal distances, in order to highlight the challenges of enforcing atypically large interpersonal distances.

**Method:**

To understand the challenges posed by social distancing requirements, we integrate relevant contributions from visual perception, social perception, and human factors.

**Results:**

To date, research on preferred interpersonal distances suggests that social distancing could induce discomfort, heighten arousal, and decrease social signaling in the short term. While the protracted effects of social distancing are unclear, we propose hypotheses on the mid- to long-term consequences of violating preferred norms of interpersonal distances.

**Conclusion:**

We suggest that enforcing a physical distance of 1.5–2 m presents a serious challenge to behavioral norms.

**Application:**

We address how notifications, architectural design, and visualizations could be effectively applied to promote interpersonal distance requirements.

Severe acute respiratory syndrome coronavirus 2 (SARS-CoV-2) is a pandemic virus that has led to the ubiquitous adoption of extreme mitigation measures. Arguably, it has presented the greatest medical, economical, and societal challenge of the 21st century. The virus is transmitted through particles emitted by the respiratory system of infected individuals and is thus primarily transmitted from person to person during social interactions (e.g., Bahl et al., 2020). To slow the spread of SARS-CoV-2, common mandatory measures include facial masks as well as keeping a minimum interpersonal distance of at least 1.5–2 m (BZgA, 2020; CDC, 2020; Chu et al., 2020). Doing so can reduce the reproduction rate of a pandemic respiratory virus by about 38% (Caley et al., 2008) and has shown a promising reduction in the reproduction rate of SARS-CoV-2 (Chu et al., 2020).

Unfortunately, this requirement for physical distance contravenes normal proxemic behavior. Typically, we maintain a smaller interpersonal distance of about 1 m when interacting with unfamiliar people (Hecht et al., 2019; Welsch et al., 2019). This preference is robust across different cultures, albeit with considerable variance (Sorokowska et al., 2017). In fact, we are willing to adopt even smaller interpersonal distances (IPD), at least for short durations, for example, when being in a crowded area (Desor, 1972). Therefore, the mandated IPD of at least 1.5 m challenges the preferred default.

To understand the challenges posed by social distancing requirements, we integrate relevant contributions from visual perception, social perception, and human factors. We review short- to long-term effects of mandated distancing on social interactions and comment on potential compliance strategies.

## Interpersonal Distance as an Expression of Personal Space

Individual distance requirements were first studied when comparing flight behavior between captive and wild animals (Heidiger, 1950). A zone is typically maintained and negotiated between animals that can be shaped by circumstances, for example, captivity. Sommer (1959) systematically investigated this need for space in human social interactions. On this basis, Hall (1966) proposed that four circular regions of egocentric space, defined by increasing radii, are distinctly reserved for social interactions: intimate space for the partner or family (0–45 cm), personal space only entered by close friends (45–120 cm), social space for interaction with strangers (120–365 cm), and public space for the general public (365–762 cm). Note that Hall’s theoretical propositions were derived from observational case studies alone. Controlled experimental studies could validate the circularity of personal space but consistently found a smaller personal space with a radius of about 1 m (e.g., Hecht et al., 2019). Functionally, this minimal distance allows us to see one another’s faces and eyes in detail while remaining at arm’s length (Hall, 1966). Intrusion into this space by a stranger often causes arousal or discomfort (Hayduk, 1978). SARS-CoV-2 has mandated larger IPD between strangers in public spaces. How will this redefine our personal space?

## Interpersonal Distance Regulation

Intrusions into our personal space can prompt us to counter-react by increasing IPD or to abort the social interaction (Felipe & Sommer, 1966). In addition, we might generate social signals to indicate a need for personal space, for example, gaze aversion, body realignment, or angry facial expression (Aranguren, 2015; Patterson, 1976; Stephenson & Rutter, 1970).

The SARS-CoV-2 requirements violate IPD expectations by demanding us to interact across unnaturally large distances. Welsch et al. (2019) obtained ratings of subjective discomfort (0 at ideal distance to 100 maximally too far or too close) for IPDs ranging from 40 to 250 cm and found that discomfort, and thus a feeling of uneasiness and distress in response to the stimulus, was consistently induced with IPDs that either intruded or exceeded personal space boundaries (~100 cm). Discomfort rose immediately when personal space was intruded. It rose linearly but at a shallower slope when IPD exceeded the comfort region of personal space (see also Thompson et al., 1979).

What determines IPD preferences in social interactions? Equilibrium theory suggests that preferred IPD arises as a compromise of approach and avoidance forces (Argyle & Dean, 1965). Thus, any deviation from this equilibrium point constitutes a violation of IPD expectations. Avoidance forces can arise from situational as well as personal variables. For example, increased IPD can be caused by arousal (Mathews et al., 1974), social threat (Vagnoni et al., 2018), stigma (Earnshaw & Quinn, 2013; Kleck et al., 1968; Neumann et al., 2004), and most importantly fear of a contagious disease (Cohn et al., 1992). Conversely, approach forces can result from a need for intimacy (Argyle & Dean, 1965) or interpersonal attraction (Welsch, von Castell, Rettenberger, et al., 2020). We conclude that the preferred IPD of about 1 m in dyadic interactions constitutes an equilibrium point of approach-avoidance forces that is stable and associated with a strong individual preference.

We rely on social signals, in particular facial expressions, to regulate an appropriate IPD (Welsch, von Castell, Hecht, 2020). Thus, the recommendation to wear facial masks in the SARS-CoV-2 crisis poses a further complication. Most people are proficient at detecting the facial expression of others (Hess et al., 2016). However, expression detection can be significantly compromised by face masks (Fischer et al., 2012) and this could result in unintended violations of IPD requirements, that is, due to people coming closer to decipher the facial expression. In a dyadic communication paradigm, angry facial expressions prompted our subjects to keep, on average, 13 cm more distance compared to a happy facial expression (Welsch, von Castell, Hecht, 2020). In an online experiment, Cartaud et al. (2020) found that the distance deemed most appropriate to a virtual person wearing a facial mask was about 12 cm smaller than that to a virtual person not wearing a mask. Thus, we may actually gravitate toward closer IPDs when interacting with others wearing facial masks.

## Interpersonal Distance Perception and The SARS-CoV-2 Crisis

Increasing the physical distance between people has been practiced in past epidemics (Caley et al., 2008), such as the SARS-CoV-1 epidemic (Lau et al., 2010) and the H1N1 epidemic (Earnshaw & Quinn, 2013). It is considered to be one of the most effective strategies to slow the spread of droplet-based or airborne viruses, once pandemic levels have been reached (Jefferson et al., 2008). However, effective adoption requires people to comprehend the need for physical distance, and adhere to it. Also, the benefits have to exceed the psychological costs of the intervention.

A larger than normal IPD, imposed by regulations, can produce a strong and continuous strain on our instinctive drive toward balancing avoidance and approach forces. This is especially demanding because IPD regulation often occurs in a fast and automatic manner (Hall, 1966; Leibman, 1970; Welsch, von Castell, Hecht, 2020). For example, IPD preferences can be predicted within 600–800 ms of social encounters, from approach-avoidance reaction times (Welsch, von Castell, Hecht, 2020, Welsch, von Castell, Rettenberger, et al., 2020). Therefore, even if people are motivated to adhere to distance requirements, lapses can occur due to the automatic processing and regulation of preferred IPDs.

## Adaptation Processes

Little is known about potential long-term adaptation to abnormal IPD that could result from the mandatory requirements of the SARS-CoV-2 crisis. Until now, proxemic research has focused on how IPD changes as an immediate response to nonverbal cues (Vagnoni et al., 2018; Welsch, von Castell, Hecht, 2020). Social isolation (Gifford & Sacilotto, 1993; Worchel, 1986; Wormith, 1984) and loneliness (Layden et al., 2018) have both been shown to increase IPD preferences. Likewise, responses to signs of disease, disgust, and other stigmata are correlated with larger IPD in social interactions (Earnshaw & Quinn, 2013; Kleck et al., 1968; Neumann et al., 2004; Toppenberg et al., 2015). Thus, if the process is reciprocal, enforcing larger IPD over long durations could induce loneliness. However, none of these effects have been investigated across long time periods.

Social interactions at enlarged distances elicit comparably high levels of discomfort (Welsch et al., 2019), probably because of the suboptimal sensory properties of the social interaction. This is in line with Hall (1966), who notes that the intensity and availability of sensory perceptions of others is a crucial factor in regulating proximity. A strong perfume (Nesbitt & Steven, 1974) or an intense gaze (Argyle & Dean, 1965) normally produce larger distances in a conversation. These cues may no longer regulate proximity when people adopt enlarged distance norms.

The efficacy of IPD as a social signal might also be compromised. For example, IPD is decreased to communicate dominance (Lobbestael et al., 2018) or group membership (Fini et al., 2020). Conversations held across a fixed distance of 1.5–2 m no longer provide these social instruments. With prolonged exposure to overly large IPD, conversations and other social processes optimized for 1 m might no longer provide equivalent utility.

We might expect more pronounced social signaling to compensate the reduced utility of proxemic cues (Argyle & Dean, 1965), such as intensified gaze, facial expression, modulation of the voice, posture, and so on. However, we are aware of only one study that provides empirical support for this (Burgoon & Aho, 1982). Here, close, normal, or large IPDs were introduced in controlled field experiments with sales personnel. Large IPDs produced shorter, less verbose, more tense, and louder sales conversations, with more distance readjustments. This would suggest that social signaling could increase, commensurate with the mandatorily increased distances, during the SARS-CoV-2 crisis. However, facial masks may render such compensation attempts futile. Thus, prolonged periods of large IPDs could give rise to alternative forms of exaggerated social signaling.

Adaptation processes have been observed on an individual level. For example, a personal crisis or traumatic event can strongly and persistently increase an individual’s need for larger IPDs (Bogovic et al., 2014). From cross-cultural proxemic studies, we know that different IPD norms can coexist. Preferred IPD, as measured by a paper-and-pencil task in 42 countries, ranged from 77 cm (Argentina) to 140 cm (Romania), Germany and the United States being in the middle with values of 96 and 95 cm, respectively (Sorokowska et al., 2017). Presumably, long-term adaptation processes are responsible for intercultural variability. Nonetheless, the SARS-CoV-2 distancing requirements of 1.5–2 m necessitate an increase of social distance far beyond the equilibrium point, even for cultures with the maximal IPD preference. We speculate that persistence of physical distance requirements will increase the likelihood of larger IPD preferences (Leibman, 1970) that could linger even after the SARS-COV2 crisis is overcome.

## Design Recommendations and Future Directions

**Figure 1:**
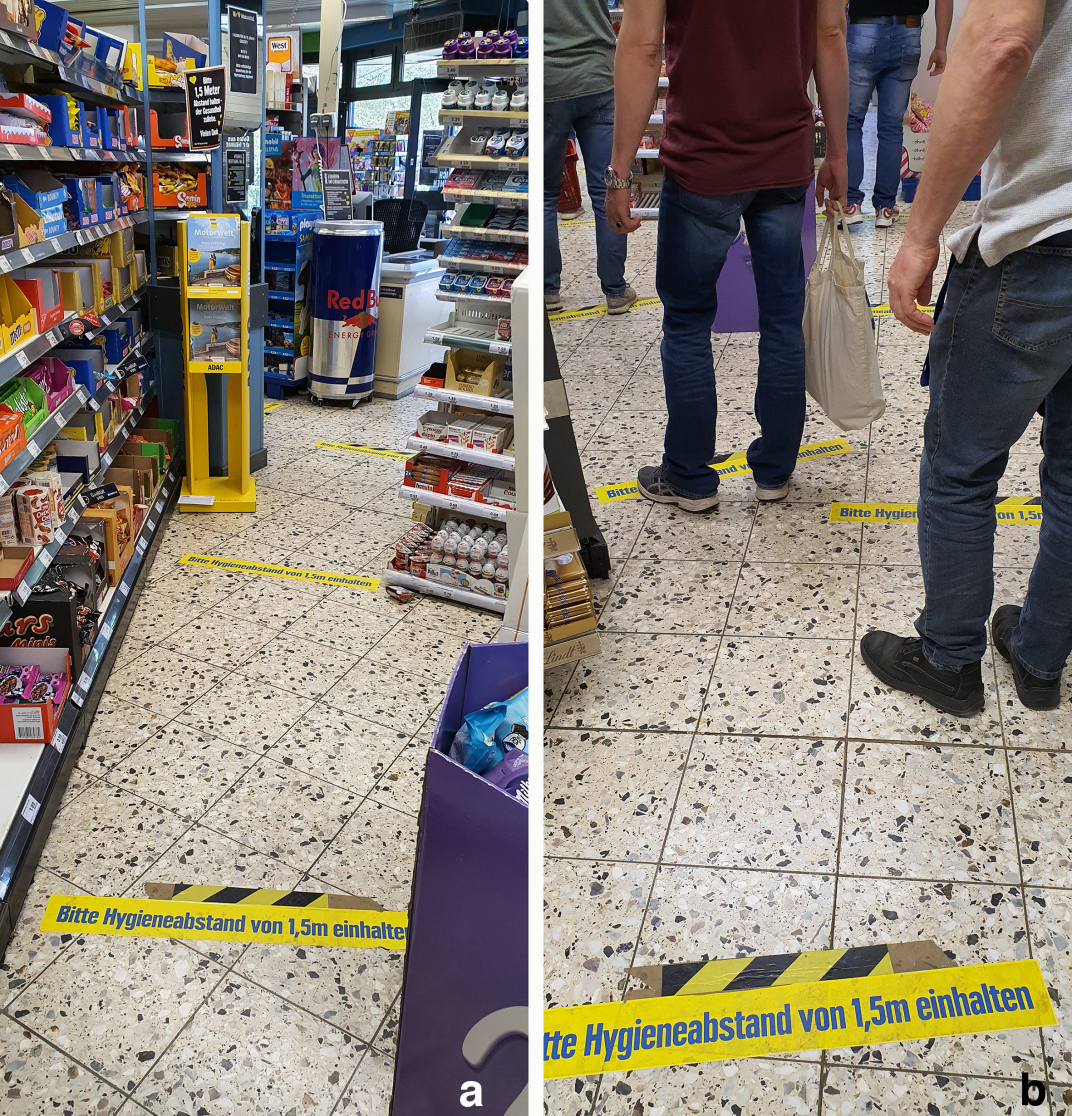
(a) Labels on the floor of a convenience store mandating a distance of 1.5 m. The yellow labels are spaced 1.5 m apart in one direction only. (b) People queuing and violating distance requirements because only frontal distances have been considered in the design of the labels.

Significant efforts have been invested to ensure adherence to the physical distance requirements of mitigating SARS-CoV-2. This includes prominent visual and auditory warnings (Figure 1a left panel for examples) as well as physical barriers, such as boxes in front of cash registers. Some countries even take punitive legal actions when physical distance requirements are flouted. Yet, many still fail to comply with physical distancing requirements. The obvious reason for this is that it contradicts to a preferred IPD of 1 m in open spaces. In fact, constrained spaces, such as the convenience store of Figure 1, typically encourage us to tolerate temporary shifts toward even closer IPD. It is possible that soft constraints, such as signs and warning sounds, could ensure higher compliance by exploiting perceptual mechanisms of distance and space estimation.

Interior design elements could distort perceptual space toward the equilibrium point of 1 m while maintaining 1.5-m physical distance. Specifically, color and lighting design could manipulate perceived social density in this fashion. Baum and Davis (1976) asked subjects to place as many figures into model rooms as would fit for “comfortable” density. They found that significantly fewer figures were placed in the room with walls that were painted a dark shade of green, compared to a light shade of green. This parallels findings that darker surface colors make rooms appear smaller compared to lighter colors (von Castell et al., 2017, 2018) and that personal space appears to be relatively smaller under highly illuminated conditions (Adams & Zuckerman, 1991).

Aside from interior design, it is documented that the room in which a person is located alters the expression of personal space in social interaction. White (1975) compared IPD in a small and a large rectangular room, and found an inverse relationship between room size and personal space size; a larger room resulted in smaller IPD, a smaller room in larger IPD. In contrast, the room size of square rooms does not seem to alter IPD (Leventhal et al., 1978). Worchel (1986) conceptually replicated these effects by comparing preferred seating distance in a small and a large room while controlling for room shape (rectangular vs. square rooms). In line with prior work, he found the size of personal space to be inversely related to room size only for the rectangular rooms, but not for square rooms. Only Cochran and Urbanczyk (1982) have so far investigated the effect of ceiling height on preferred IPD. A room with a low ceiling as compared to the same room with a higher ceiling produced a preference for larger IPDs. Therefore, we suspect that when carefully considering the architectural properties of a room, IPD can be enlarged to some extent and thus encourage social distancing.

Mere alterations of architecture, color, and illumination are unlikely to be solely sufficient to enforce an IPD of 1.5 m, given the strong attractor forces toward an IPD of 1 m. However, they can make compliance more bearable. Ultimately, they are unlikely to substitute physical barriers, such as transparent sneeze screens. Interestingly, Desor (1972) demonstrated that such transparent barriers can foster acceptance of increased social density. Interior design measures that reduce perceived distance and/or allow for safe decreased physical distance should be considered as a complement to cognitive reminders of the unnatural distance requirements.

In sum, we suggest that enforcing a physical distance of 1.5–2 m presents a serious challenge to behavioral norms. This distance requirement exceeds the preferred social distance in encounters with strangers; it is almost double the accepted social norm. Physical distancing requirements push our social interactions beyond the equilibrium point that balances numerous implicit forces, which promote avoidance and approach between individuals. Here, we propose several testable hypotheses regarding short- and long-term effects of keeping enlarged IPD. In the short term, we suspect that people will feel discomfort when having a conversation and that social signaling may be increased to allow for efficient communication. In the long term, we expect new social signaling patterns to emerge and the norm for IPD to enlarge due to an adaption process. Finally, we recommend exploiting perceptual heuristics to reduce perceived IPD, and to allow for natural IPD with transparent physical barriers to meet the big challenge, namely, to reduce the spread of the virus while allowing for social encounters at the comfortable IPD of about 1 m.

## Key Points

- Enforcing a physical distance of 1.5–2 m presents a serious challenge to behavioral norms.
- The currently mandated minimum social distance is almost double the accepted social norm of 1 m.
- Social distancing could induce discomfort, heighten arousal, and limited social signaling in the short-term.
- In the long term, we hypothesize that new social signaling patterns may emerge to compensate enlarged distances and that personal space may be persistently enlarged due to an adaption process.
